# Effect of arterial blood bicarbonate (HCO_3_^−^) concentration on the accuracy of STOP-Bang questionnaire screening for obstructive sleep apnea

**DOI:** 10.1186/s12890-021-01720-2

**Published:** 2021-11-13

**Authors:** Chong Pei, Shuyu Gui

**Affiliations:** 1 Department of Respiratory and Critical Care Medicine, The First Affiliated Hospital of Anhui Medical University, No. 81 of Mei Shan Road, Hefei, 230001 Anhui People’s Republic of China; 2 Department of Respiratory and Critical Care Medicine, The Third Affiliated Hospital of Anhui Medical University (The First People’s Hospital of Hefei), No. 390 of Huai He Road, Hefei, 230001 Anhui People’s Republic of China

**Keywords:** Obstructive sleep apnea, Arterial blood HCO_3_^−^ concentration, Polysomnograms, STOP-Bang questionnaire, Epworth Sleepiness Scale, Berlin questionnaire

## Abstract

**Background:**

To evaluate the effect of arterial bicarbonate (HCO_3_^−^) concentration on the accuracy of STOP-Bang questionnaire (SBQ) screening for obstructive sleep apnea (OSA).

**Methods:**

A total of 144 patients with suspected OSA were included. Polysomnograms (PSG) and blood gas analysis were performed, and the Epworth Sleepiness Scale (ESS), STOP-Bang questionnaire, and Berlin questionnaire were completed. The correlation between the arterial HCO_3_^−^ concentration, apnea hypopnea index (AHI), and other related indicators was analyzed. The scoring results of the ESS, SBQ, and Berlin questionnaire were compared with the PSG results, and the sensitivity and specificity were calculated in the form of a four-cell table. The changes in the sensitivity and specificity of OSA screening after SBQ alone and combined with HCO_3_^−^ concentration were compared, and ROC curves were drawn.

**Results:**

Arterial HCO_3_^−^ concentration was positively correlated with AHI (r = 0.537, P < 0.001). The ratio of HCO_3_^−^ concentration ≥ 24.6 mmol/L in the non-OSA group was significantly lower than that in the OSA group (25.0% VS 80.8%, P < 0.001). The sensitivity of the SBQ was higher than that of the ESS (97.5% VS 81.7%, P < 0.001) and the Berlin questionnaire (97.5% VS 79.2%, P < 0.001). There was no statistical significance in the specificity of the three scales (25%, 37.5%, 37.5%). A combined SBQ score ≥ 3 and HCO_3_^−^ concentration ≥ 24.6 mmol/L showed increased specificity and decreased sensitivity compared with an SBQ score ≥ 3 alone, with a corresponding AUC of 0.771 (P < 0.01) and 0.613 (P > 0.05), respectively.

**Conclusion:**

The sensitivity of the SBQ was better than that of the Berlin questionnaire and ESS. After combining arterial blood HCO_3_^−^ concentration, the SBQ questionnaire increased the specificity of OSA prediction and decreased the sensitivity, which improved the accuracy of screening.

**Supplementary Information:**

The online version contains supplementary material available at 10.1186/s12890-021-01720-2.

## Background

Obstructive sleep apnea (OSA), also known as obstructive sleep apnea hypopnea syndrome (OSAHS), is characterized by repeated apnea and hypopnea during sleep [1]. With an estimated 425 million individuals affected worldwide, OSA poses a global public health problem [2]. Polysomnography monitoring (PSG) is the gold standard for the diagnosis of OSA, but the examination is time-consuming and expensive. Most primary hospitals in China are not equipped with PSG equipment, and many patients have to wait for a long time for an appointment. Further, some questionnaire results are inconsistent with the gold standard, which delays the diagnosis and treatment of real OSA patients. It is particularly important to adopt simple methods for preliminary screening and diagnosis of OSA. The Berlin Questionnaire, STOP-Bang Questionnaire (SBQ), and Epworth Sleepiness Scale (ESS) are widely used in OSA screening and have good sensitivity [3, 4]. In general, the screening efficiency of these questionnaires is not ideal. Some OSA patients also have chronic daytime hypercapnia [5]. To maintain the acid–base balance in the body, the kidney reduces the excretion of HCO_3_^−^, leading to an increase in arterial blood HCO_3_^−^. The combination of HCO_3_^−^ and a questionnaire is hypothesized to improve the sensitivity or specificity of questionnaires alone [6]. The purpose of this current study is to improve the screening efficiency of OSA through the combination of arterial blood HCO_3_^−^ and a questionnaire, to facilitate the diagnosis and treatment of OSA patients earlier in future clinical work. This study compared the difference between the SBQ, the Berlin questionnaire, and the ESS screening for patients with OSA, and examined the effect of arterial HCO_3_^−^ concentration on SBQ screening.

## Methods

### Patient selection

This prospective study was approved by the institutional ethics committee of the Third Affiliated Hospital of Anhui Medical University, and informed consent was obtained from all patients to include their data in this study. Patients who underwent PSG examination at the Sleep Center of the Third Affiliated Hospital of Anhui Medical University (First People's Hospital of Hefei) from March 2019 to October 2020 were screened for study inclusion. Inclusion criteria were: (1) age > 18 years old; (2) complete autonomous behavior and cognitive ability; (3) arterial blood gas analysis and PSG monitoring performed during hospitalization; and (4) ability to answer the questionnaire completely and accurately. Exclusion criteria were: (1) associated diseases such as chronic obstructive pulmonary disease, acute attacks of asthma, interstitial pulmonary disease, laryngospasm, vocal cord paralysis, tracheal foreign bodies, anemia, and electrolyte disorders that may affect blood gas analysis; (2) other underlying diseases that may affect HCO_3_^−^ concentration, such as liver, kidney, lung, and heart dysfunction; (3) use of a ventilator in the past three months; (4) other common sleep respiratory disorders besides OSA; (5) pregnancy, lactation, puerperium, and mental disorders (including a history of depression or anxiety); (6) use of sedatives or antipsychotic treatment over the past three months; (7) patients with an abnormal EEG and (8) if patients are using diuretics.

### PSG recording and analysis

All subjects were monitored using an Alice6 PSG instrument (Philips Respironics, USA) and analyzed manually by sleep technicians to confirm or exclude OSA. PSG was performed with 16 channels, including: electrooculography, electroencephalography, electrocardiography, electromyography (submental and bilateral tibial), airflow measurements with both oronasalthermal sensors and nasal air pressure transducers, transtracheal sounds via microphone, rib cage and abdominal movement by inductance plethysmography using thoracoabdominal belts, and continuous pulse oximetry. Studies were scored using the 2012 American Academy of Sleep Medicine (AASM) [7] scoring guidelines with hypopneas scored as a 30% drop in the nasal pressure from baseline for at least 10 s and associated with either arousal or drop in oxygen saturation by 3%. The apnea hypopnea index (AHI), lowest blood oxygen saturation (LSaO_2_) and mean blood oxygen saturation (MSaO_2_) were recorded. OSA was diagnosed according to the Chinese Guidelines for Primary Diagnosis and Treatment of Adult Obstructive Sleep Apnea (2018). These include (1) clinical symptoms of any one or more of the following: daytime sleepiness, non-recovery of energy after waking, fatigue, or insomnia; waking up because of breathlessness, poor breathing, or suffocation at night; habitual snoring and breathing interruption; and hypertension, coronary heart disease, stroke, heart failure, atrial fibrillation, type 2 diabetes, mood disorders, or cognitive impairment; (2) PSG monitoring: AHI ≥ 5 times/h, mainly obstructive events; (3) none of the above symptoms and PSG monitoring: AHI ≥ 15 times/h, mainly obstructive events. Adult OSA can be diagnosed if criteria (1) and (2) are met or only criterion (3) is met. Grade of disease: AHI: 5–15 times/h is mild, > 15–30 times/h is moderate, and > 30 times/h is severe. The percentage of total sleep time when blood oxygen is less than 90% (CT90%), duration of apnea hypopnea in total sleep time (AHT%), mean apnea-hypopnea duration (MAD), and duration of apnea hypopnea per hour (HAD) were calculated based on the reported data. PSG is the gold standard for the diagnosis of OSA.

### Blood gas analysis

Two milliliters of radial artery blood were collected with a special syringe for blood gas analysis (American Westmed). Samples were obtained from study subjects following PSG monitoring and when they were awake and seated in a quiet room. The sample was mixed and sealed, and blood gas analysis was performed within 30 min to detect HCO_3_^−^. The process of specimen extraction strictly followed the requirements of aseptic operation.

### ESS

The ESS asks individuals to grade their sleepiness during several routine activities including sitting and reading, watching TV, sitting in a public place, a long ride (more than 1 h), talking with people, resting after dinner (not drinking), driving, and reposing at rest in the afternoon. Each condition is divided into four grades: never (0), rarely (1), sometimes (2) and often (3). The subjects will score according to their own conditions, and the researchers will calculate the total score. An ESS score ≥ 9 was associated with high risk of OSA, and an ESS score < 9 was associated with a low risk of OSA (shown in Additional file 1: Fig. 1).

**Fig. 1:**
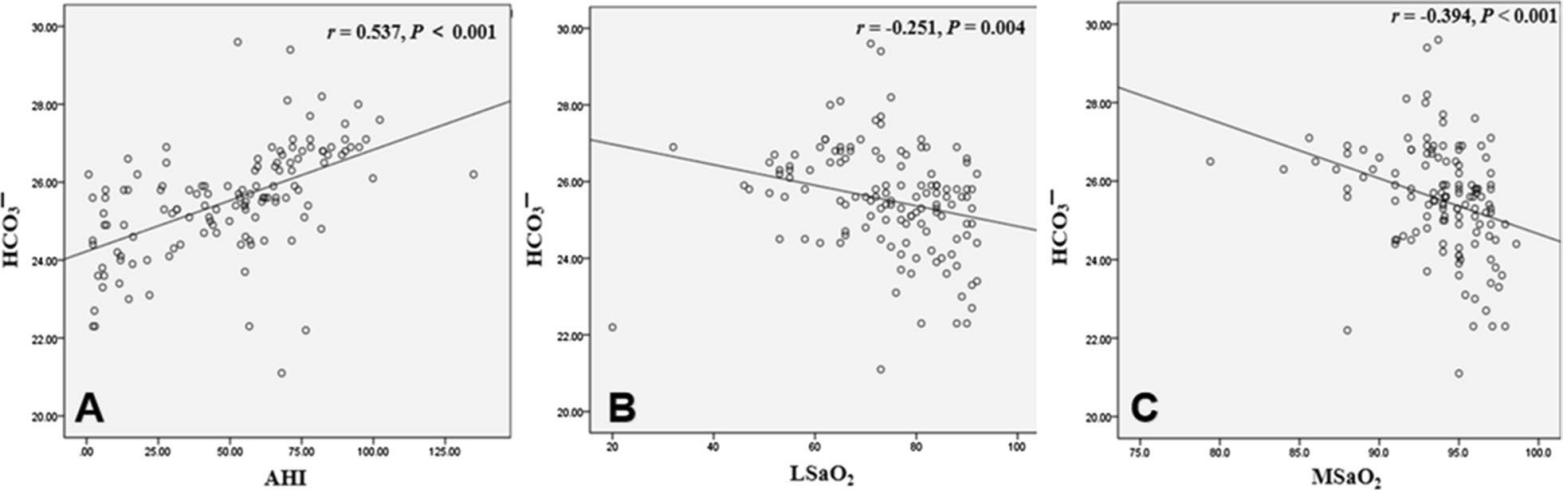
**A** HCO_3_^−^ concentration is positively correlated with AHI (*r* = 0.537, *P* < 0.001); **B** HCO_3_^−^ concentration is negatively correlated with LSaO_2_ (*r* =  − 0.251, *P* = 0.004); **C** HCO_3_^−^ concentration is negatively correlated with MSaO_2_ (*r* =  − 0.394, *P* < 0.001)

### SBQ

The questionnaire comprised eight characteristics: S, snoring; T, tiredness; O, observed apnea; P, high blood pressure; B, body mass index > 35 kg/m^2^; A, older than 50 years; N, neck circumference > 40 cm; and G, male sex. Patients answered the first four questions (STOP questions), and the staff in the sleep room measured the height, weight, blood pressure, and neck circumference of the patients. Then, the respondents answered the last four questions (BANG questions). The answer was yes (1 points) or no (0 points), and the total score was calculated. A score ≥ 3 was defined as a high risk of OSA, while a score < 3 was defined as a low risk of OSA (shown in Additional file 2: Fig. 2).

**Fig. 2:**
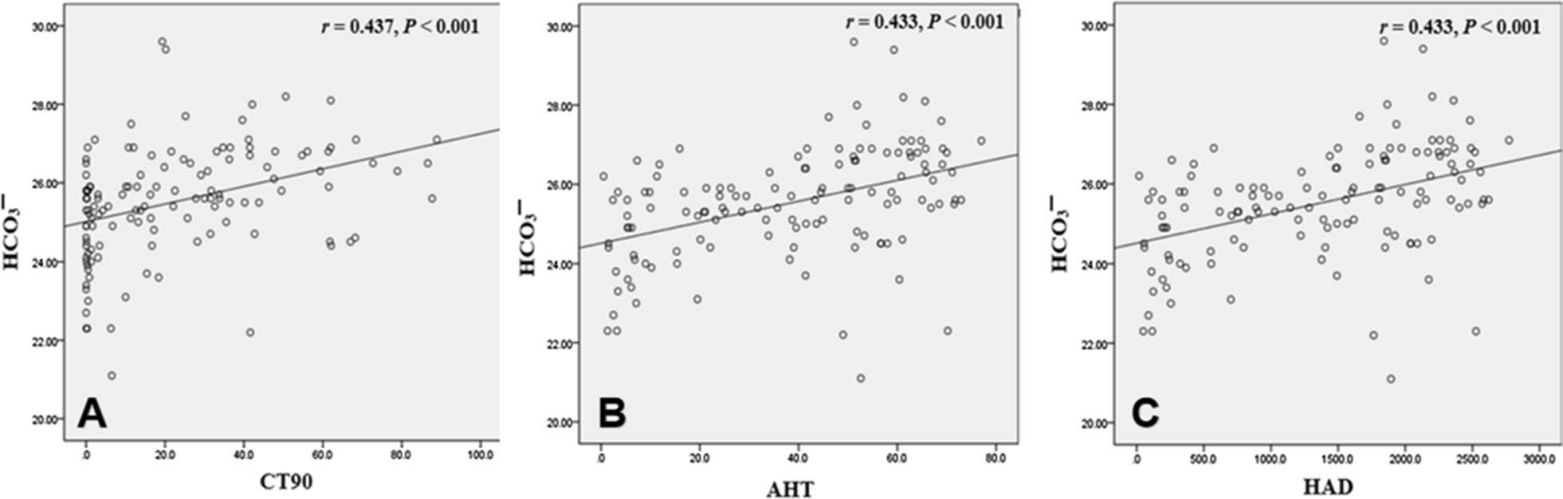
**A** HCO_3_^−^ concentration is positively correlated with CT90 (*r* = 0.437, *P* < 0.001); **B** HCO_3_^−^ concentration is positively correlated with AHT (*r* = 0.433, *P* < 0.001); **C** HCO_3_^−^ concentration is positively correlated with HAD (*r* = 0.433, *P* < 0.001)

### Berlin questionnaire

The Berlin questionnaire consists of ten questions plus information on height and weight arranged in three categories: snoring and cessation of breathing (category 1; five questions); symptoms of excessive daytime sleepiness (category 2; four questions); and BMI and hypertension (category 3; one question and height and weight information). Some of the questions had to be answered by the patient's family members or co-residents to ensure the accuracy of the answers. Positive scores in 2 or more categories suggest that the respondent is at high risk for OSA (shown in Additional file 3: Fig. 3).

**Fig. 3:**
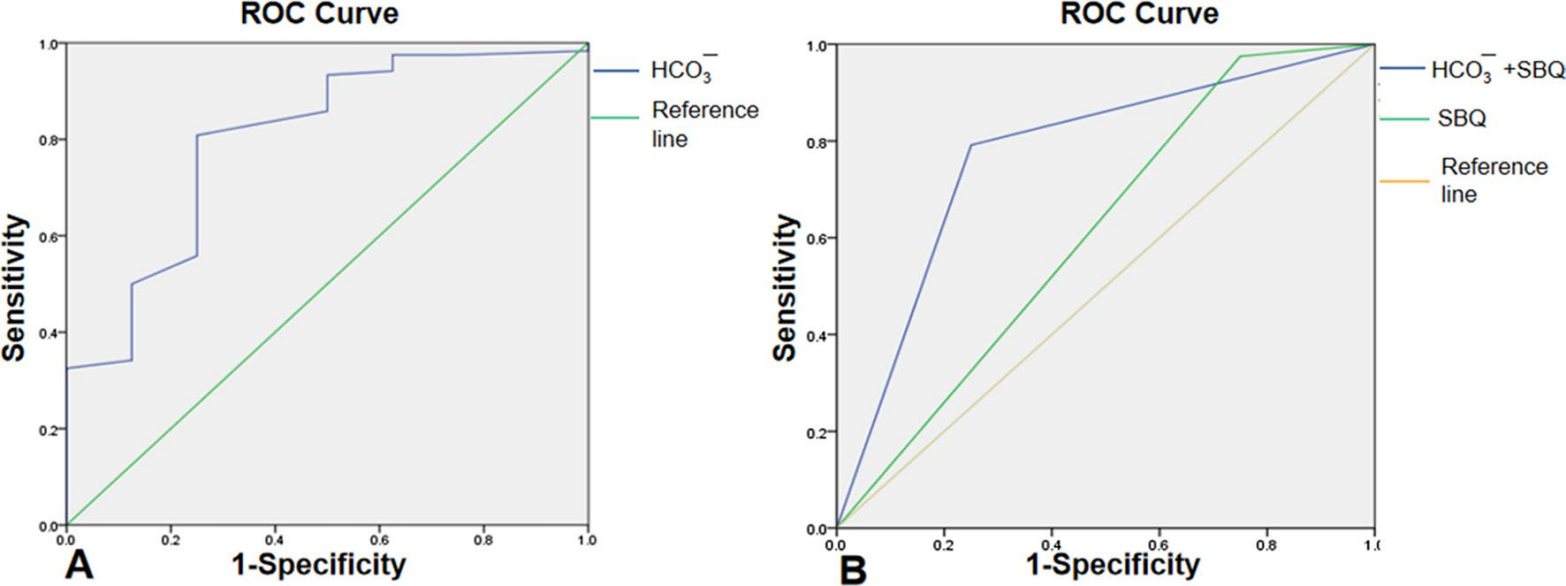
**A** HCO_3_^−^ concentration predicts OSA as demonstrated by the corresponding area under the ROC curve (AUC) being 0.80; **B** a combination of the SBQ questionnaire score and arterial HCO_3_^−^ concentration is more predictive of OSA than either alone. Corresponding AUC of the OSA group was 0.613 (*P* > 0.05). When the combined SBQ score was ≥ 3 and the HCO_3_^−^ concentration was ≥ 24.6 mmol/L, the corresponding AUC in the OSA group increased to 0.771 (*P* < 0.01)

### Analytical methods

All patients received PSG monitoring, blood gas analysis, and completed the ESS, SBQ, and Berlin questionnaires. According to AHI and symptoms, the patients were divided into the non-OSA group or the OSA group, and the latter group was further divided into mild (5 times/h ≤ AHI ≤ 15 times/h), moderate (15 times/h < AHI ≤ 30 times/h), and severe (AHI > 30 times/h). Patients were divided into two groups according to the SBQ: the high-risk OSA group (STOP-Bang questionnaire score ≥ 3 points) and low-risk OSA group (STOP-Bang questionnaire score < 3 points). Patients were divided into two groups according to ESS: ESS ≥ 9 was classified as high-risk for OSA, and ESS < 9 was classified as low-risk for OSA. According to the Berlin questionnaire, the patients were divided into two groups: positive for the high-risk OSA group and negative for the low-risk OSA group. A correlation analysis between HCO_3_^−^ concentration and AHI, LSAO2, MSAO2, CT90%, AHT%, MAD, and HAD was carried out to judge the correlation degree, and the diagnostic value of HCO_3_^−^ concentration on OSA was analyzed and evaluated. The sensitivity and specificity of the three kinds of questionnaires for screening OSA were compared. Then, the diagnostic value of the STOP-Bang questionnaire score alone and SBQ combined with the HCO_3_^−^ concentration for OSA was further compared.

### Statistical methods

SPSS19.0 statistical software was used for data analysis. Patient information was analyzed by descriptive statistics. The normal distribution data is represented by $\stackrel{-}{X\hspace{0.17em}}$± s, and the non-normal distribution data is represented by the median (M) and the interquartile range (P75–P25). Correlation analysis was conducted between the HCO_3_^−^ concentration and AHI, CT90%, AHT%, MAD, and HAD, and the correlation coefficients were calculated. Then, calculation was done for the degree of sensitivity, specific diagnostic test evaluation of the four tables, and sensitivity to the specific degrees of comparison between different methods using the matching χ2 test (with P < 0.05 for the difference being statistically significant). The optimal truncation value of the HCO_3_^−^ concentration for the diagnosis of OSA was analyzed by Youden index. A ROC curve was used to compare the scores of SBQ alone and the diagnostic value of SBQ combined with HCO_3_^−^ concentration for OSA.

## Results

### General information

The general morphological data of 144 patients with suspected diagnosis of OSA are shown in Table 1. According to the PSG monitoring results, the 144 patients with suspected diagnosis were divided as follows: 24 in the non-OSA group and 120 in the OSA group, including 17 mild cases, 12 moderate cases, and 91 severe cases.

**Table 1:** Demographic and physiologic data of the study cohort

Parameters	OSA	non-OSA
*Sex*		
Male	108	15
Female	12	9
Age (years)	45.0 ± 12.9	39.3 ± 15.7
Neck circumference (cm)	41.9 ± 3.7	37.4 ± 3.7
BMI (kg/m^2^)	27.7, 4.8	24.9 ± 4.9
AHI	52.9 ± 27.0	2.4 ± 0.9
CT90 (%)	17.0, 38.0	0.1, 0.3
AHT (%)	40.2 ± 21.7	2.0, 1.7
MAD (s)	27.5 ± 7.5	26.5, 9.1
HAD (s)	1446.7 ± 781.0	72.7, 58.5
HCO_3_^−^ (mmol/L)	25.6 ± 1.3	24.0 ± 1.4
PaCO_2_ (mmHg)	40.6 ± 3.7	38.0 ± 3.5
LSaO_2_ (%)	73.2 ± 13.0	90.0, 3.8
MSaO_2_ (%)	94.1, 3.0	97.0 ± 1.1
SBQ	5.0, 1.0	3.0, 3.0
ESS	13.8 ± 4.9	9.1 ± 5.2
*Berlin Questionnaire*		
Positive	95	15
Negative	25	9

BMI = body mass index, AHI = apnea hypopnea index, CT90% = percentage of total sleep time when blood oxygen is less than 90%, AHT% = duration of apnea hypopnea in total sleep time, MAD = mean apnea–hypopnea duration, HAD = duration of apnea hypopnea per hour, LSaO_2_ = lowest blood oxygen saturation, MSaO_2_ = mean blood oxygen saturation, SBQ = STOP-Bang questionnaire, ESS = Epworth Sleepiness Scale

### Correlation between HCO_3_^−^ concentration and PSG index and its predictive value for OSA

The HCO_3_^−^ concentration was positively correlated with AHI (r = 0.537, P < 0.001) (Fig. 1A), negatively correlated with LSaO_2_ (r =  − 0.251, P = 0.004) (Fig. 1B), negatively correlated with MSaO_2_ (r =  − 0.394, P < 0.001) (Fig. 1C), positively correlated with CT90 (r = 0.437, P < 0.001) (Fig. 2A), positively correlated with AHT (r = 0.433, P < 0.001) (Fig. 2B), positively correlated with HAD (r = 0.433, P < 0.001) (Fig. 2C), and had no correlation with MAD.

When the HCO_3_^−^ concentration was applied to predict OSA, the corresponding area under the ROC curve (AUC) was 0.80 (Fig. 3A), sensitivity was 80.1% and specificity was 75%. After calculating the corresponding Yoden index, the HCO_3_^−^ concentration of 24.6 mmol/L had the best cutoff value for screening (Yoden index = 0.558). The ratio with an HCO_3_^−^ concentration ≥ 24.6 mmol/L in the non-OSA group was significantly lower than that in the OSA group (25.0% vs. 80.8%, P < 0.001). The ratio with an HCO_3_^−^ concentration ≥ 24.6 mmol/L in the severe OSA group was significantly higher than that in the mild OSA group (87.9% vs. 52.9%, P = 0.001), while there was no statistically significant difference in the ratio between the severe OSA group and the moderate OSA group (87.9% vs. 66.7%, P = 0.127).

### Comparison of sensitivity and specificity of the SBQ, ESS and Berlin questionnaires

Sensitivity, specificity, positive predictive value (PPV), and negative predictive value (NPV) of the three questionnaires for predicting OSA are shown in Table 2.

**Table 2:** Diagnostic efficiency of three questionnaires for predicting OSA

Parameters	Sensitivity (%)	Specificity (%)	PPV (%)	NPV (%)
SBQ	97.5	25.0	86.7	66.7
ESS	81.7	37.5	86.7	29.0
Berlin	79.2	37.5	86.4	26.5

SBQ = STOP-Bang questionnaire, ESS = Epworth Sleepiness Scale, PPV = positive predictive value, NPV = negative predictive value

The sensitivity of the SBQ questionnaire compared with the ESS questionnaire (97.5% VS 81.7%, P < 0.001) was statistically significant. The sensitivity of the SBQ questionnaire compared with the Berlin questionnaire (97.5% vs. 79.2%, P < 0.001) was also statistically significant. There was no statistically significant difference in sensitivity between the ESS and Berlin questionnaires (81.7% vs. 79.2%, P = 0.701).

There was no statistical significance in the specificity of the three scales (25%, 37.5%, 37.5%).

### The value of SBQ alone and SBQ combined with HCO_3_^−^ concentration screening for OSA

The sensitivity and specificity of all OSA, moderate and severe OSA, and severe OSA were calculated, with an SBQ score ≥ 3 as the screening cutoff value, as shown in Table 3. A combined SBQ score ≥ 3 points and HCO_3_^−^ concentration ≥ 24.6 mmol/L showed increased specificity and decreased sensitivity (Table 3).

**Table 3:** Values of SBQ ≥ 3 points combined with an HCO_3_^−^ concentration ≥ 24.6 mmol/L for screening for OSA (%)

Cutoff value	AHI ≥ 5	AHI ≥ 15	AHI ≥ 30
SBQ ≥ 3 points			
Sensitivity	97.5	98.1	98.9
Specificity	25.0	17.1	15.1
SBQ ≥ 3 points + HCO_3_^−^≥24.6 mmol/L			
Sensitivity	79.2*	84.5*	86.8*
Specificity	75.1*	65.9*	58.5*

AHI = apnea hypopnea index, SBQ = STOP-Bang questionnaire

Annotation: *Compared with the SBQ score ≥ 3 group, P < 0.01

When the SBQ was applied alone to predict OSA, the corresponding AUC of the OSA group was 0.613 (P > 0.05). When a combined SBQ score ≥ 3 and the HCO_3_^−^ concentration ≥ 24.6 mmol/L, the corresponding AUC in the OSA group increased to 0.771 (P < 0.01) (Fig. 3B).

## Discussion

Various examinations and questionnaires exist to evaluate the degree of sleepiness and quality of life to diagnosis OSA. However, none of them can completely satisfy clinical needs. While more accurate, some methods are complex and inconvenient such as PSG, which is the gold standard for the diagnosis of OSA [1, 8]. The SBQ, ESS, and Berlin questionnaires are commonly used in the clinical evaluation of OSA and are less expensive and more convenient than PSG [1, 9, 10].

SBQ is a self-evaluation questionnaire developed by Canadian investigators [11] and sleep medicine experts in 2008. The questionnaire is based on the STOP questionnaire, and includes the common symptoms of OSA (snoring, fatigue, sleep apnea, high blood pressure, BMI, sex, age, and neck circumference) and adopts "yes/no" questions as a second grading method. It is simple to use, can be done in a shorter time, and is easily accepted by subjects. The questionnaire was used for screening OSA patients preoperatively in the sleep clinic with high sensitivity [11, 12]. In keeping with this, we found the SBQ questionnaire more sensitive than the ESS and Berlin questionnaires. However, the specificity of the three scales was equally low.

Chronic daytime hypercapnia (PaCO_2_ ≥ 45 mmHg) is observed in 10% to 38% of patients with OSA [13]. This result is similar to the findings of our study. There were 10 patients with PaCO2 ≥ 45 mmHg in our study, and all of them were in the OSA group, accounting for 8.3%. Interval nocturnal hypercapnia due to obstructive apnea or hypopnea may result in renal HCO_3_^−^ retention to compensate for respiratory acidosis; this may lead to the elevation of serum HCO_3_^−^ [6].

In our study, compared with the SBQ questionnaire alone, the specificity and sensitivity of the SBQ combined with arterial blood HCO_3_^−^ concentration in screening OSA were significantly increased and decreased, respectively. However, by calculating and comparing the AUC, we found that the diagnostic accuracy of the SBQ combined with arterial blood HCO_3_^−^ concentration was significantly better than that of the SBQ questionnaire alone. A serum HCO_3_^−^ of at least 28 mmol/L and an SBQ score of at least 3 were found to improve the specificity of the prediction of moderate to severe OSA, but sensitivity was reduced [6, 14]. The results of these studies on the effect of serum HCO_3_^−^ on SBQ sensitivity and specificity are similar to ours. However, we found that the value of HCO_3_^−^ was quite different from our current research results. In the current study cohort of 144 patients, only 3 cases (2.1%) had an HCO_3_^−^ concentration ≥ 28 mmol/L, and the optimal cutoff value of the HCO_3_^−^ concentration was 24.6 mmol/L. A correlation between the BMI and the severity OSA was reported [15] Another study found that as the severity of OSA increases, the risk of chronic daytime hypercapnia may also increase [16].This finding is relevant, as the BMI of Asians is generally lower than that of Europeans and Americans [17], and may, in part, explain the low HCO_3_^−^ concentration cutoff value in our study.

In addition, our current study confirmed a significant correlation between AHI and HCO_3_^−^ concentration; this has also been reported in previous studies [6, 18]. We also found a certain correlation between the LSaO_2_, MSaO_2_, CT90, AHT, HAD, and HCO_3_^−^ concentration.

Improvements and modification of the various questionnaires continue to be pursued. This is especially true of the SBQ questionnaire. Except for HCO_3_^−^ concentration, BMI may be a focus of modification in relation to the scoring, but the results were inconsistent [19, 20]. Some scholars believe that the cut-off value of BMI in the STOP-BANG questionnaire is 28 kg/m^2^, which is more suitable for the Chinese population than other questionnaires [21]. However, the modified STOP-B28(STOP + BMI > 28 kg/m^2^) questionnaire had the same high sensitivity and low specificity as the original SBQ in screening for OSA [22].

## Conclusions

In conclusion, the present study indicates that Chinese individuals with a SBQ score ≥ 3 and an arterial HCO_3_^−^ concentration ≥ 24.6 mmol/L should be considered as having OSA, and therefore PSG monitoring should be performed to confirm the diagnosis. This approach is expected to be helpful in more precisely identifying those individuals in which the more intensive technique of PSG will be useful. The combination of the SBQ questionnaire and arterial blood HCO_3_^−^ concentration showed good diagnostic performance.

There are several limitations to the present study. First, the subjects of this study were all patients who went to the sleep center already with a suspected diagnosis of OSA, and therefore the non-OSA group was relatively small; thus, there may have been a selection bias. Second, the time of arterial blood collection was the morning after the completion of PSG, and the proportion of patients diagnosed with severe OSA was relatively high, which may have affected the results. Third, the study included predominantly male participants, limiting its application to the female population. In addition, home sleep tests were not included in this study. This is mainly due to the inability to conduct a more comprehensive examination in the outpatient clinic to determine whether a patient meets the inclusion criteria, as does blood gas analysis. Because in our country most patients need to bear their own outpatient expenses, which is a difficulty.

## Supplementary Information

**Additional file 1: Fig. 1.** Epworth Sleepiness Scale (ESS).

**Additional file 2: Fig. 2.** STOP-Bang Questionnaire (SBQ).

**Additional file 3: Fig. 3.** Berlin Questionnaire.

## Data Availability

The data will be available upon reasonable requests. Shuyu Gui (Email: 117913380@qq.com) should be contacted if someone wants to request the data.

## Abbreviations

SBQ: STOP-Bang questionnaire
OSAHS: Obstructive sleep apnea hypopnea syndrome
OSA: Obstructive sleep apnea
PSG: Polysomnograms
ESS: Epworth Sleepiness Scale
AHI: Apnea hypopnea index
LSaO_2_: Lowest blood oxygen saturation
MSaO_2_: Mean blood oxygen saturation
CT90%: Percentage of total sleep time when blood oxygen is less than 90%
AHT%: Duration of apnea hypopnea in total sleep time
MAD: Mean apnea-hypopnea duration
HAD: Duration of apnea hypopnea per hour
