# Supplementary figures and images for: Effect of arterial blood bicarbonate (HCO3−) concentration on the accuracy of STOP-Bang questionnaire screening for obstructive sleep apnea
Source: BMC Pulm Med. 2021 Nov 13;21:366. doi: 10.1186/s12890-021-01720-2 (PMC8590281; doi:10.1186/s12890-021-01720-2)

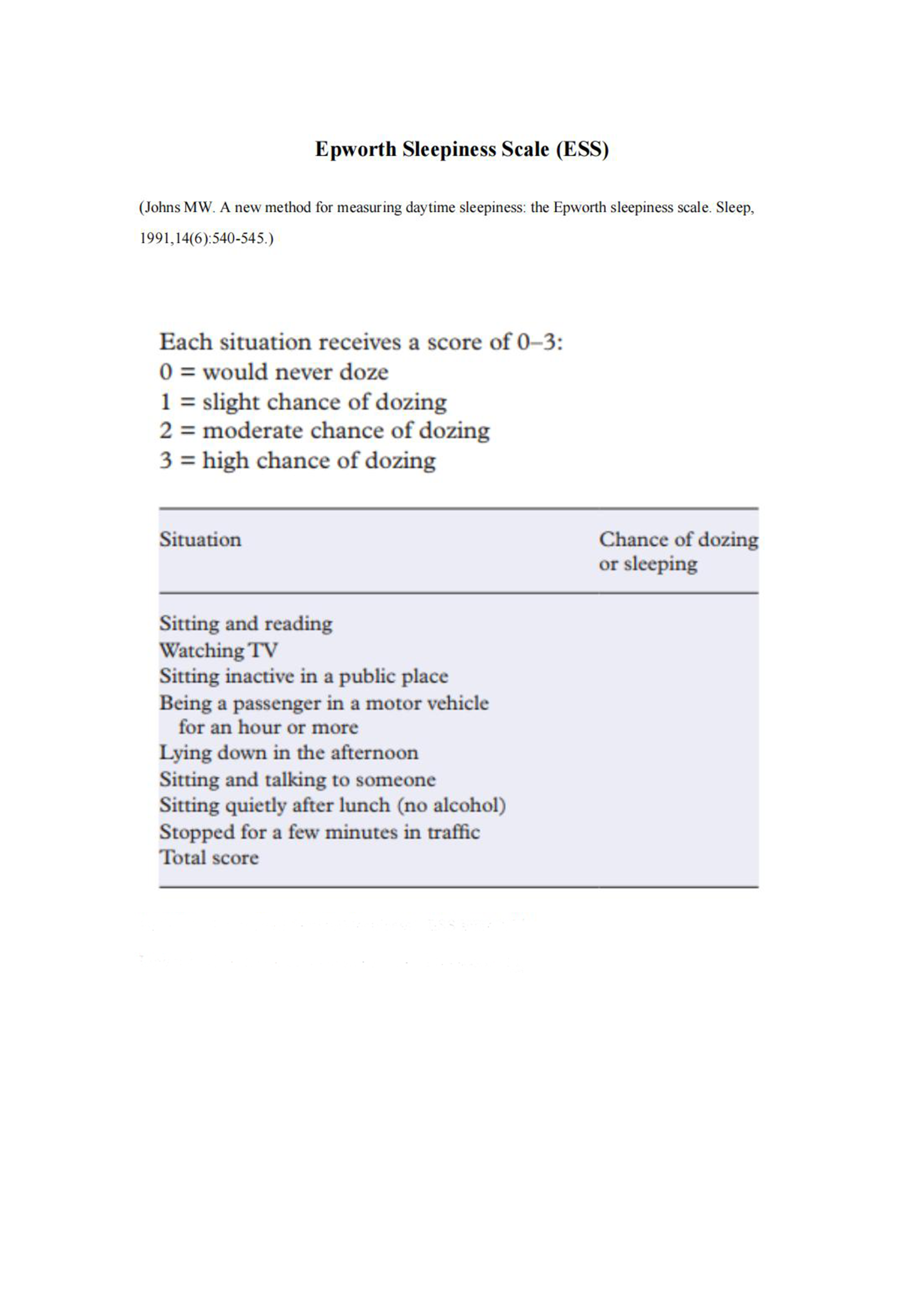

Supplement: Supplementary file 1 — Additional file 1: Fig. 1. Epworth Sleepiness Scale (ESS). [file 12890_2021_1720_MOESM1_ESM.tif]

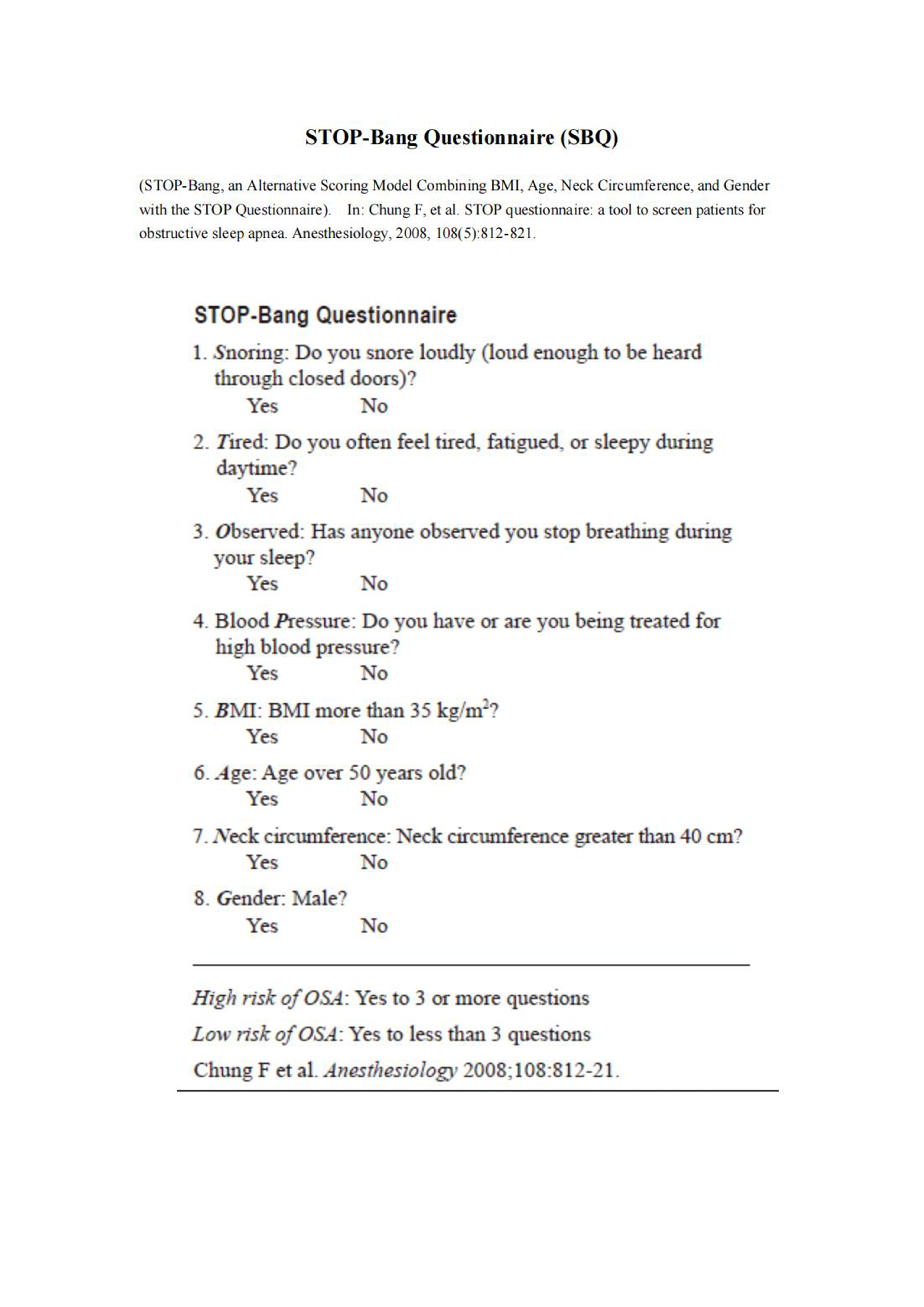

Supplement: Supplementary file 2 — Additional file 2: Fig. 2. STOP-Bang Questionnaire (SBQ). [file 12890_2021_1720_MOESM2_ESM.tif]

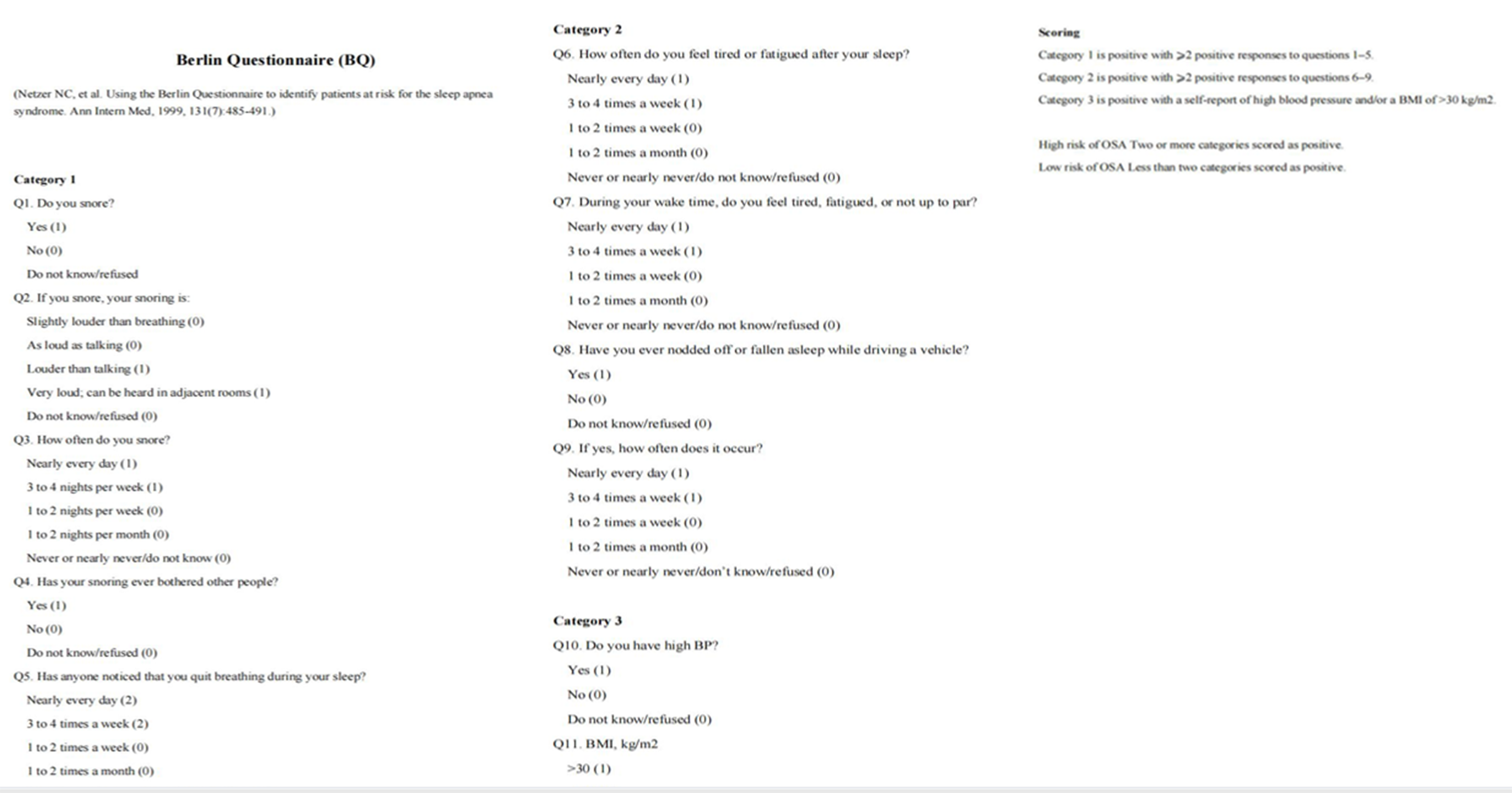

Supplement: Supplementary file 3 — Additional file 3: Fig. 3. Berlin Questionnaire. [file 12890_2021_1720_MOESM3_ESM.tif]
